# AI-driven clustering and visualization of electrocardiogram signals to enhance screening for atrial fibrillation: The supermarket/hypermarket opportunistic screening for atrial fibrillation study

**DOI:** 10.1016/j.hroo.2025.07.003

**Published:** 2025-07-12

**Authors:** Ryan A.A. Bellfield, Pablo Rendon Hormiga, Ivan Olier, Robyn Lotto, Ian Jones, Gregory Y.H. Lip, Sandra Ortega-Martorell

**Affiliations:** 1 Data Science Research Centre, Liverpool John Moores University, Liverpool, United Kingdom; 2 Liverpool Centre for Cardiovascular Science at University of Liverpool, Liverpool John Moores University and Liverpool Heart and Chest Hospital, Liverpool, United Kingdom; 3 Escuela Colombiana de Ingeniería Julio Garavito, Bogota, Colombia; 4 School of Nursing and Advanced Practice, Liverpool John Moores University, Liverpool, United Kingdom; 5 Danish Center for Health Services Research, Department of Clinical Medicine, Aalborg University, Aalborg, Denmark

**Keywords:** Generative topographic mapping, Artificial intelligence, Machine learning, Atrial fibrillation, Screening, Detection

## Abstract

**Background:**

Atrial fibrillation (AF) is the most common arrhythmia worldwide, associated with an increased risk of serious health issues. As its prevalence rises, health care systems face significant challenges, including escalating treatment costs and the inherent difficulties of detecting AF, particularly in paroxysmal cases where symptoms are intermittent.

**Objective:**

This study investigates the application of unsupervised machine learning, specifically generative topographic mapping (GTM), to support AF screening and risk stratification.

**Methods:**

The supermarket/hypermarket opportunistic screening for atrial fibrillation study deployed single-lead electrocardiogram (ECG) sensors (MyDiagnostick) embedded in supermarket trolley handles across 4 sites in Northwest England. This community-based approach successfully engaged the public in opportunistic AF screening. However, diagnosis was limited by reliance on transient ECG recordings. To improve analysis, we selected a subset of 97 ECG traces (78 for training and 19 for testing) reviewed by a consultant cardiologist, comprising AF (n = 23), possible AF (n = 9), and normal rhythm (n = 65). From these, 477 20-second ECG snippets were extracted to train the GTM model.

**Results:**

The GTM generated interpretable membership maps, clustering ECG snippets into visually distinct regions with similar features. These maps enable clinicians to explore heart rhythm dynamics over time and track patient trajectories across risk states.

**Conclusion:**

This study demonstrates the potential of our proposed methodology to uncover latent patterns in ECG data, providing deeper insights into individual heart rhythm patterns and supporting more nuanced AF risk assessment and the overall effectiveness of AF detection and management. By embedding interpretable artificial intelligence in screening tools, we aimed to improve early detection and reduce the clinical burden of AF.

**Key Findings**

▪ Innovative screening approach: we developed a novel method using single-lead electrocardiographic (ECG) data from sensors embedded in the handles of supermarket trolleys for the opportunistic screening of atrial fibrillation (AF), the most common arrhythmia worldwide, associated with an increased risk of serious health issues.
▪ Application of generative topographic mapping (GTM): we used GTM, an unsupervised artificial intelligence (AI) and machine learning (ML) technique, to develop and probabilistic model and generate interpretable “membership maps” for the visualization of ECG signal patterns.
▪ Utilization of a unique dataset: as part of the supermarket/hypermarket opportunistic screening for atrial fibrillation study, we recorded 118 single-lead ECGs, producing 477 ECG snippets that were subsequently used to train the GTM model.
▪ Enhanced visual interpretability: we generated interpretable visualizations that cluster ECG snippets with similar features, enabling clinicians to analyze patient trajectories and monitor transitions between distinct cardiac rhythm states.
▪ Clinical relevance: we demonstrated that AI-driven membership maps can facilitate real-time identification of individuals at risk of AF, providing a transparent and accessible tool for detecting subtle variations in heart rhythm.
▪ Community-based implementation: we highlighted the feasibility of using AI/ML methodologies for opportunistic AF screening in community settings, enhancing public engagement with health technology.
▪ Advances in AF management: we provided evidence that integrating unsupervised AI/ML techniques into AF detection can support personalized management strategies, addressing the challenges of identifying paroxysmal and asymptomatic cases.

## Introduction

Atrial fibrillation (AF) is the most common arrhythmia disorder worldwide, with its presence being associated with a higher risk of strokes, heart failure, and other conditions.^1^ Additional challenges related to AF include the increasing cost of treatment, a burden expected to intensify as the prevalence of AF continues to rise in the coming years.^2^ The detection of AF is inherently challenging, particularly in cases of paroxysmal AF, where the arrhythmia manifests intermittently. Diagnosis frequently relies on the arrhythmic episodes being recorded on an electrocardiogram (ECG) during a monitoring period, complicating detection unless the individual presents with persistent AF.^3^

One approach to mitigating this issue is through systematic screening, a process that aims to identify the likelihood of disease in individuals who appear asymptomatic. This method facilitates the early detection of potential conditions, enabling timely medical intervention that may not have been sought otherwise.^4^^,^^5^ Studies such as SAFE,^6^ STROKESTOP,^7^ LOOP,^8^ SEARCH-AF,^9^ and GUARD-AF^10^ have demonstrated the effectiveness of various AF screening methods, reporting enhanced detection rates and reduced adverse clinical outcomes.

Despite these advances, several reviews of different AF screening methods have been unable to support widespread implementation, primarily owing to the limited number of randomized controlled trials^11^ showing inconsistencies in study designs, which hinder meaningful comparisons.^12^ In addition, the effectiveness of traditional screening in health care settings or through pharmacy interventions may be constrained by several factors. For example, individuals from lower socioeconomic groups are often less likely to engage with health care professionals, and there is a perceived inconvenience for asymptomatic individuals, who may be less motivated to participate in screening initiatives.^3^^,^^13^

The supermarket/hypermarket opportunistic screening for atrial fibrillation (SHOPS-AF) study aimed to assess the feasibility of using an embedded MyDiagnostick single-lead ECG sensor in the handles of shopping trolleys in 4 supermarkets in Northwest England.^13^ These provided a single-lead ECG recording for AF screening in the community as people shopped in supermarkets that contained resident pharmacists.^13^ The study successfully demonstrated that the public is willing to engage with AF screening when it is conveniently integrated into their regular routine. It also yielded promising results in detecting AF among participants.

Artificial intelligence (AI), more specifically machine learning (ML), has been used in screening programs to assist health care professionals in assessing an individual’s risk of the targeted disease. In the context of AF screening, AI/ML has been primarily used to develop predictive models aimed at a more accurate and efficient detection of AF.^14^, ^15^, ^16^, ^17^, ^18^ These models have demonstrated significant potential to enhance screening accuracy through techniques such as convolutional neural networks (CNNs). By minimizing the need for human intervention, these approaches could facilitate the screening of a larger population, thereby increasing overall detection rates of AF.

AI/ML has also been used to optimize AF screening. The study by Adeniji et al^19^ used multiple logistic regression to prioritize which ECGs should be reviewed, thereby reducing the overall number of ECGs requiring screening. Their approach demonstrated a reduction in workload while maintaining AF detection accuracy. However, both methods have their limitations.

For example, AI/ML approaches that use “black box” models, such as CNNs, may not yield interpretable results,^20^ potentially undermining health care professionals’ trust in the algorithm’s efficacy. Second, some of the studies listed earlier attempt to incorporate a degree of interpretability, using methods such as gradient-weighted class activation maps (Grad-CAM).^17^ However, these techniques are applied post hoc and offer only a limited insight into the model’s decision-making processes. Grad-CAM can also be prone to erroneously highlighting areas of the input as important,^21^ which further contributes to the distrust associated with this approach. Another common theme across all the aforementioned studies is their reliance on supervised learning techniques.

Unsupervised learning methods (also under the umbrella of AI/ML) offer significant advantages over traditional binary predictions by uncovering hidden patterns and structures within data without requiring labeled training examples, thereby facilitating the discovery of new subgroups and clinically relevant phenotypes.^22^^,^^23^ In addition, this approach enables more nuanced data representation through techniques such as clustering and dimensionality reduction, enhancing interpretability and deepening the understanding of complex datasets.

In this study, we aimed to develop an unsupervised AI/ML methodology that provides interpretable visualizations to assist clinicians in screening for AF. This objective will be achieved using generative topographic mapping (GTM), a probabilistic ML technique that compresses data into a lower-dimensional space. In particular, we will build upon the methodology outlined by Bellfield et al^23^ to enhance the delineation of groups generated by the model.

## Methods

### Data description

#### Data description and selection

The data used within this study are a subset taken from the dataset collected as part of the SHOPS-AF study.^13^ The embedded MyDiagnostick medical device in the handles of supermarket trolleys recorded a single-lead ECG tracing for a participant as they held on to the handle while shopping. The device illuminated a red light if the participant’s ECG was thought to be AF. When AF was detected, these participants were reviewed by a pharmacist who performed a manual pulse reading and referred the participant to a consultant cardiologist for further cardiac assessment.

**Figure 1:**
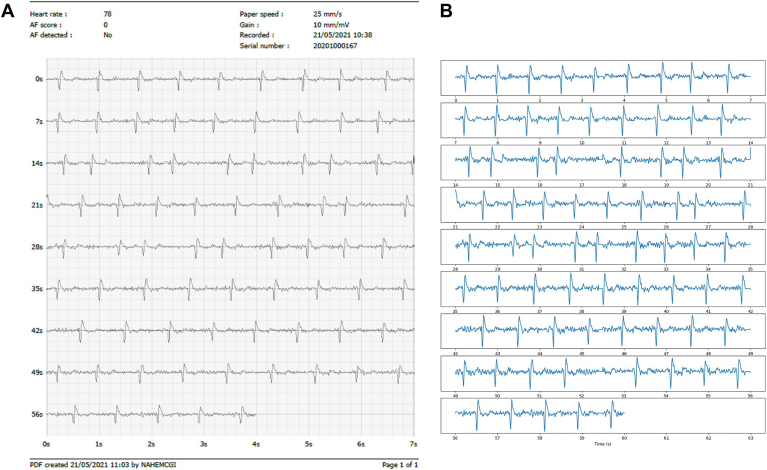
**A:** Displays the data in the PDF format directly collected from the medical device. **B:** Displays the digitized waveforms extracted from the PDFs that were used in the analysis. AF = atrial fibrillation.

The data collected by the medical device are stored as PDFs, as shown in Figure 1A. The data subset used for this analysis consisted of ECGs collected from SHOPS-AF that were selected after the study to be reviewed by a consultant cardiologist to confirm the diagnosis of the ECG to give a ground truth, whether this be sinus rhythm or AF, as opposed to using the reading from the medical device as the sole diagnosis.

#### Data preprocessing

Given that the ECGs were stored in PDF format, we opted to use the raw signals instead of the PDFs for 2 primary reasons. First, our analysis focused on features extracted from the ECG signals, which are considerably easier to obtain from the raw data than from image files. Second, existing literature indicates that using extracted signals, particularly when recordings are sufficiently long, is more effective for ML applications than relying on images of ECG signals.^21^ To digitize the signal, we applied the waveform extraction methodology outlined by Bellfield et al^21^ to extract every “row” of the ECG from the PDF, which were then stitched together to form 1 long ECG waveform. Examples of the digitized signals are presented in Figure 1B.

**Figure 2:**
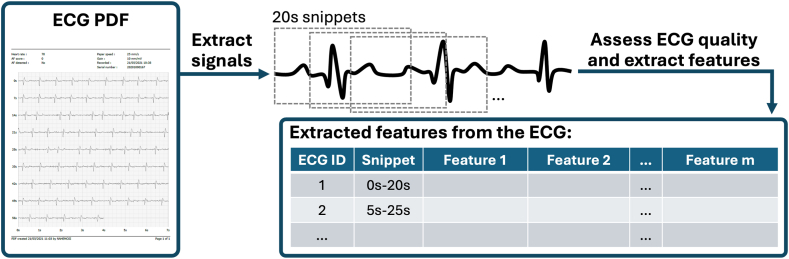
Outlines the workflow to go from the original ECG in PDF format to the extracted features that will be used for model development. ECG = electrocardiogram.

Once the signal was extracted, we capped its length at 60 seconds and divided the waveform into multiple snippets using a sliding window of 20 seconds with a 75% overlap (5-second stride length), as illustrated in Figure 2. This method generated a maximum of 9 snippets for each ECG signal. There were several reasons for adopting this approach: first, it serves as a form of data augmentation to enhance the dataset size, a common strategy for optimizing ML models during training. Second, and more critically, AF may not be present throughout the entire ECG signal, often occurring only in specific segments. By splitting the ECG into snippets, we can isolate the regions that exhibit AF.

Once the snippets were generated, we assessed their quality to ensure that they were sufficiently robust to yield meaningful clinical insights. To this aim, we used the “Neurokit2” Python package^24^ to automatically evaluate the quality of the snippets, excluding any that were not classified as excellent from further analysis.

Then, key features were extracted from the signals in each snippet relating to various aspects of the heart variability of the ECG, again using the NeuroKit2 package. The full workflow is illustrated in Figure 2. We then applied a correlation threshold of 0.9 to remove highly correlated variables and all variables with a missingness percentage of >30% to achieve the final modeling dataset. The list of variables used for this analysis is presented in Table 1.

**Table 1:** Details all the heart variability variables extracted from the ECG signals using the Neurokit2 package that were included for model development

Variable type	Variable name	Variable description
Time domain	HRV_MedianNN	RR interval – median
	HRV_MadNN	RR interval – mean absolute deviation
	HRV_Prc20NN	RR interval – 20th percentile
	HRV_Prc80NN	RR interval – 80th percentile
	HRV_pNN50	RR interval – absolute difference % above 50 ms
	HRV_pNN20	RR interval – absolute difference % above 20 ms
	HRV_MinNN	RR interval – minimum
	HRV_HTI	HRV triangular index
	HRV_TINN	RR interval distribution approximation
Frequency domain	HRV_HF	High-frequency spectral power
	HRV_HFn	Normalized high frequency
HR fragmentation	HRV_PIP	Percentage of inflection points of the RR intervals series
	HRV_PSS	Percentage of short segments
	HRV_PAS	Percentage of RR intervals in alternation segments
Characteristics of the Poincaré plot geometry	HRV_SD1SD2	The ratio between short- and long-term fluctuations of the RR intervals
	HRV_CSI	CSI
	HRV_CVI	Cardiac vagal index
	HRV_CSI_Modified	Modified CSI obtained by dividing the square of the longitudinal variability by its transverse variability
	HRV_GI	Guzik’s index
	HRV_PI	Porta’s index
	HRV_C1d	Contributions of HR decelerations to short-term HRV
	HRV_SD1d	Short-term variance of contributions of decelerations
	HRV_C2d	Contributions of HR deceleration to long-term HRV
	HRV_SD2d	Long-term variance of contributions of decelerations
	HRV_SD2a	Long-term variance of contributions of accelerations
	HRV_Cd	Total contributions of HR decelerations to HRV
DFA and multifractal DFA	HRV_DFA_alpha1	Monofractal DFA of the HR signal, corresponding to short-term correlations
	HRV_MFDFA_alpha1_Width	Width of the singularity spectrum, which quantifies the degree of multifractality
	HRV_MFDFA_alpha1_Peak	Value of the singularity exponent corresponding to the peak of the singularity dimension
	HRV_MFDFA_alpha1_Mean	Multifractal DFA – mean of singularity exponents
	HRV_MFDFA_alpha1_Max	Mean of the maximum and minimum values of the singularity exponent
	HRV_MFDFA_alpha1_Delta	Vertical distance between the singularity spectrum where the singularity exponents are at their minimum and maximum
	HRV_MFDFA_alpha1_Asymmetry	The asymmetric ratio corresponds to the centrality of the peak of the spectrum.
	HRV_MFDFA_alpha1_Fluctuation	The h-fluctuation index
Indices of complexity	HRV_ApEn	The approximate entropy measure of HRV
	HRV_ShanEn	Shannon entropy
	HRV_FuzzyEn	Fuzzy entropy of a signal
	HRV_CD	Correlation dimension – a lower-bound estimate of the fractal dimension of a signal
	HRV_HFD	Higuchi’s fractal dimension
	HRV_KFD	Katz’s fractal dimension
	HRV_LZC	Lempel-Ziv complexity

Please refer to the Neurokit2 documentation for further information.^24^

CSI = cardiac sympathetic index; DFA = detrended fluctuation analysis; ECG = electrocardiogram; HR = heart rate; HRV = heart rate variability.

#### Ethical considerations

The ethical review has been granted by Liverpool John Moores University’s University Research Ethics Committee. The study was undertaken in compliance with the research protocol. During phase 1, verbal consent was obtained upon recruitment, with written consent secured for those with an abnormal sensor recording whose personal data would be required for onward referral for 12-lead ECGs. For the qualitative substudy (phase 2), written consent was obtained from all participants. The research adheres to relevant ethical guidelines.

### Clustering methodology

GTM

The methodology developed as part of this study uses, at its core, the GTM probabilistic data clustering unsupervised ML algorithm designed for clustering and analyzing data.^25^^,^^26^ GTM works in a latent space, often set to be 2-dimensional (2D), which helps simplify and visualize the data. The latent grid consists of a uniform grid of nodes, and the algorithm assumes that the data we observe (for our study, features extracted from the ECG snippets) are generated by mapping this simplified space onto the higher-dimensional data space where the original data exist. This mapping is achieved using the function in equation 1:

(1) y=WΦ(u)

where u is a point in the L-dimensional latent space, W is a matrix containing parameters that control the mapping, and Φ consists of S basis functions $\Phi_{S}$, which for the standard GTM (used in this study) are radially symmetric Gaussians. For the full details on how the methodology is derived, please refer to the original publications.^25^^,^^27^

In practice, GTM calculates the probability that each set of features (eg, features extracted from the ECG snippets) belongs to each node in the latent space. The data are then assigned to the node with the highest probability, a process called “mode projection.” This approach reduces the risk of unrelated data points being grouped together. Given that the latent space is 2D, the results can be displayed as a 2D map, referred to as the “membership map.”

An important advantage of GTM is that each node in the latent space corresponds to a reference vector in the original data space. These reference vectors serve to summarize the key characteristics of the data assigned to each node. This allows the user to interpret how specific features influence data assignment to different nodes. As a result, the methodology provides valuable insights into the relationships between features and patterns in the data.

#### GTM magnification factors

In an ideal scenario, latent nodes generated as part of the GTM approach that are closer together will be mapped to points that are close together in the data space, ensuring that data that share similar characteristics reside in the same area of the membership map. However, when using a latent space whereby the nodes lie on a uniform grid, areas of the latent space may be distorted to optimize the fit of the manifold to the data space. This in turn can result in the visualization of the membership map not fully capturing the actual separations as seen in the data space.

One solution to this, proposed by the original developers of GTM, is to use the concept of magnification factors.^28^ Magnification factors are derived using techniques taken from differential geometry. As previously mentioned, GTM defines a smooth mapping from a latent space to the original data space using the function defined in equation 1. The magnification factor describes how the area of an infinitesimal rectangle at a point in the latent space, for example, one of the predefined nodes, transformed to infinitesimal volumes in the higher-dimensional data space.^29^ For the full derivation, please refer to the original publication.^28^ What this translates to is that higher magnification factors show areas of high distortion during the projection, which corresponds to areas where data are sparse, with the reverse being true for lower magnification values. These can be assessed visually by superimposing the magnification factors onto the membership map generated from GTM and using a gray color scale to represent the values of the factors

#### Macrocluster generation using magnification factors

Let us consider for a moment that the nodes in the GTM latent space are microclusters of the original dataset. These are useful because they provide a way of visualizing the key underlying relationships within the data. However, one drawback is that these visualizations of microclusters may be too granular for practical use.^30^ Several studies have proposed approaches to further aggregate these microclusters into macrocluster partitions that can be interpreted more easily.^23^^,^^29^^,^^30^

The methodological contribution of this paper focuses on extending the original macroclustering methodology outlined by Bellfield et al.^23^ They first used GTM to assign data to nodes in the latent space. Then, agglomerative hierarchical clustering was performed on the reference vectors, which were subsequently mapped to their corresponding latent nodes to generate the macroclusters. Our contribution focuses specifically on improving the macrocluster generation aspect of this methodology to provide more accurate, better-defined aggregations. We do this by implementing a constrained hierarchical clustering of the reference vectors to preserve the latent node neighborhoods within macrocluster boundaries. Although constrained hierarchical clustering has previously been applied to GTM macrocluster generation, as in Vellido et al,^29^ who used a simple neighborhood constraint that included all immediate surrounding nodes while restricting merges with non-neighboring nodes, our approach introduces a more flexible and data-driven definition of neighborhoods.

**Figure 3:**
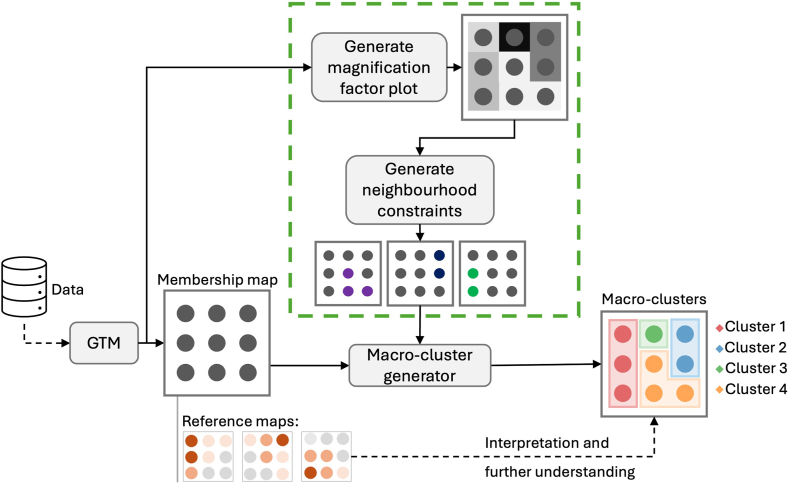
Proposed artificial intelligence–based methodology that builds on the approach in 23. The modification to the approach is contained within the *green dashed box* and demonstrates how the magnification factors are generated from the GTM model, used to identify neighborhood constraints, which are then used to influence the hierarchical clustering applied to the reference vectors. GTM = generative topographic mapping.

In particular, the novelty of our method lies in first defining neighborhoods in the latent space using magnification factors and unsupervised k-nearest neighbors (KNN), rather than relying on a fixed topological constraint. Given a set of points $U=\left\{u_{1},u_{2},\cdots ,u_{l}\text{,}\right\}$ in $R^{n}$, unsupervised KNN defines, for every point $u_{i}$, the KNNs based on the smallest distance.^31^^,^^32^ In our case, each point $u_{i}$ resides in $R^{3}$, defined by the latent node coordinates and the magnification factor calculated at that point. Euclidean distance was used to evaluate the distance between pairs of points. KNN was set to look for the 5 closest neighbors, enabling the construction of nuanced neighborhoods that incorporate the local information encoded by the magnification factors.^31^ Once neighborhoods were defined for each latent node, they were mapped to their corresponding reference vectors in the original data space. These neighborhoods were then used as constraints in the hierarchical clustering process. From this point, the methodology follows that of Bellfield et al^23^ to generate the macroclusters in the latent space, with the full process detailed in Figure 3.

#### Leveraging macroclusters for ECG screening

GTM calculates the probability of each data point (ie, features extracted from each ECG snippet) belonging to each latent node, formally known as responsibilities. Considering the responsibilities instead of the mode projections allows us to consider the full probability distribution of the data in the latent space. For our use case, each ECG will be represented by a nonzero number of snippets extracted from the longer signal. Averaging the responsibilities for all snippets relating to an ECG provides the probability distribution for the entire ECG. Once obtained, for each macrocluster, the total responsibility for all nodes assigned to it can be calculated. In practice, this means that, for an ECG, we see which macrocluster best represents that ECG and assign it to that cluster, in a very similar way to mode projection. This is useful because we can analyze the key characteristics of each macrocluster to understand what defines each of them, an approach used previously to define clinically relevant AF phenotypes,^23^ identify differences in censorship between countries,^33^ and market segmentation.^29^ By understanding which diagnostic class best represents each macrocluster, this can then be assigned to the ECG and used alongside the visualizations to help inform the clinician performing the screening.

## Results

### Membership map and reference vectors

Applying the data preprocessing steps to the clinically reviewed subset of ECGs from the full SHOPS-AF dataset, we had a total of 617 ECG snippets taken from 97 ECGs, where 477 snippets (from 78 ECGs) were used for training the GTM model with 140 snippets (from 19 ECGs) being used to validate the GTM macroclusters. Of the 78 training ECGs, 21 were diagnosed as AF, 8 were diagnosed as possible AF, and the remaining 49 were diagnosed as normal sinus rhythm, henceforth referred to as “normal.” For ECGs used for testing, 2 were diagnosed as AF, 1 as possible AF, and the remaining 16 as normal. Of the 2 ECGs reserved for testing, 1 was diagnosed as AF, and the other as normal. For the parameters of the GTM model, we used an 8 × 8 latent space, 9 radial basis functions arranged in a 3 × 3 grid, a regularization term of 1, and a width parameter of 0.8.

**Figure 4:**
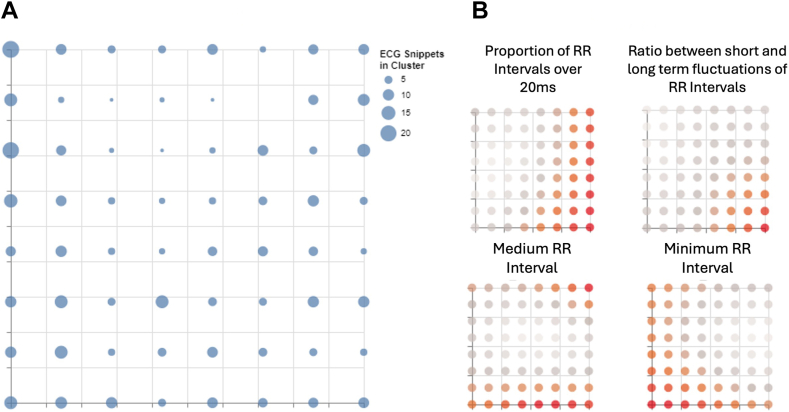
**A:** Membership map representing the latent space generated from the generative topographic mapping model trained on the features extracted from the athlete’s ECG rhythm strips. **B:** A selection of reference map visualizations demonstrating how 4 of the variables used to train the generative topographic mapping affect the cluster distribution in the latent space. ECG = electrocardiogram.

The membership map generated by the GTM when trained on the 477 ECG snippets using these set parameters is presented in Figure 4A. This map visualizes the latent space containing a compressed representation of the entire original data space. Each point on the map represents a node containing at least 1 ECG snippet, with the size of the point indicating the number of ECG snippets in the node: the larger the node, the more snippets have been assigned to it.

A selection of the reference maps extracted from the GTM model trained on the features extracted from the 477 ECG snippets (all reference maps are contained in Supplemental Figure 1 in the Supplementary Material) is presented in Figure 4B. These maps display how each variable affected what latent node an ECG snippet would be assigned to, allowing the user to interpret the model’s decision making. Each point within the reference maps corresponds to the node in the exact location in the membership map. The color scheme for the reference maps was chosen so that the points that are redder highlight that ECG snippets assigned to that node had a higher value for that variable. On the flip side, the grayer a point is, the ECG snipper assigned to the node had a lower value for that variable. For example, using the top left plot in Figure 4B, we can see that ECG snippets with a higher proportion of RR intervals of >20 ms were assigned to nodes on the right-hand side of the membership map.

### Magnification factors and macroclusters

#### Magnification factors

**Figure 5:**
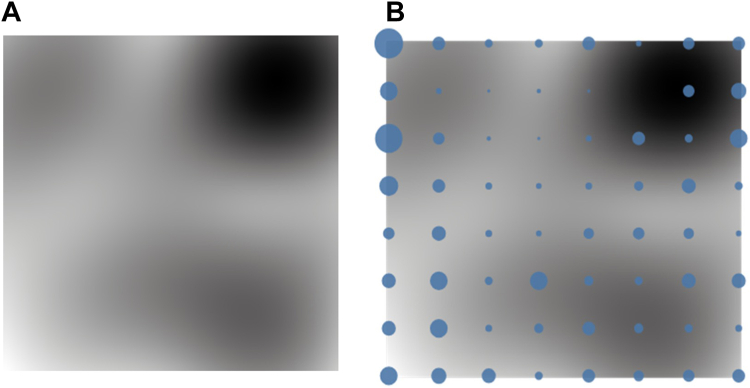
Magnification map calculated from the trained generative topographic mapping. *Light areas* of this map correspond to areas of low distortion during mapping, with the *darker areas* relating to areas of high distortion during the mapping.

The magnification factor plot generated from the trained GTM, as described in section 2.2.2, using a gray-scale representation, is presented in Figure 5. This visualization provides a representation of how the latent manifold distorts when projected and fitted to the data. Lighter areas of this visualization demonstrate the regions with low distortion in the mapping, with the darker gray corresponding to regions that experienced high distortion in the same mapping.

#### Macroclusters

**Figure 6:**
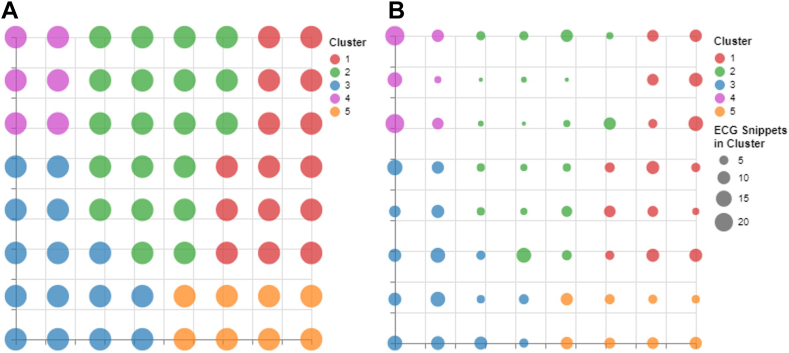
Derived subgroups of athletes using data extracted from their ECG snippets. **A:** Membership map with a uniform size for the microclusters to show the distribution of the macrocluster regions. **B:** The size of the microclusters in the membership map, dictated by the number of ECG snippets assigned to it. ECG = electrocardiogram.

**Figure 7:**
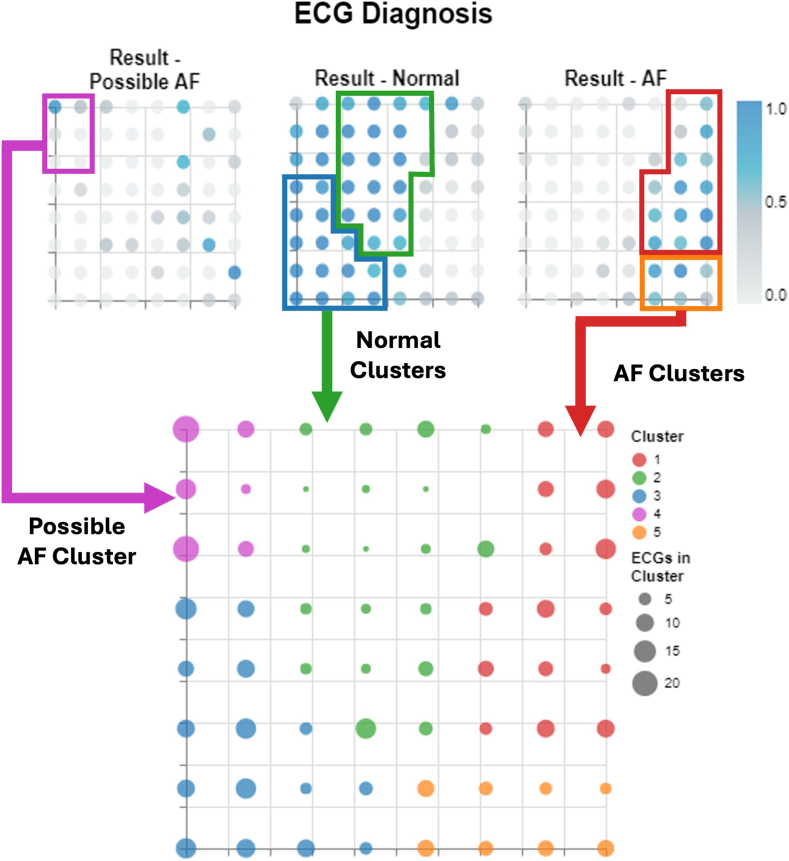
The top 3 plots display the ECG diagnoses overlaid on the membership map. The bluer a region is, the larger the percentage of ECG snippets in each node that came from an ECG diagnosis with the outlined condition. AF = atrial fibrillation; ECG = electrocardiogram.

By applying the methodology defined in this study, we were able to generate 5 macrocluster regions within the GTM latent space, as shown in Figure 6A and 6B. Each macrocluster contains nodes and by extension ECG snippets that share similar characteristics. These clusters can also be further analyzed by looking at the distribution of diagnoses within the latent space, as shown in Figure 7. From these visualizations, we can derive definitions for each of the clusters: clusters 1 and 5 relate to AF ECG snippets, clusters 2 and 3 relate to normal ECG snippets, and cluster 4 relates to possible AF ECG snippets.

The top 3 plots in Figure 7 show how the different macroclusters relate to the 3 different diagnoses present within the dataset.

### Validating screening effectiveness

**Figure 8:**
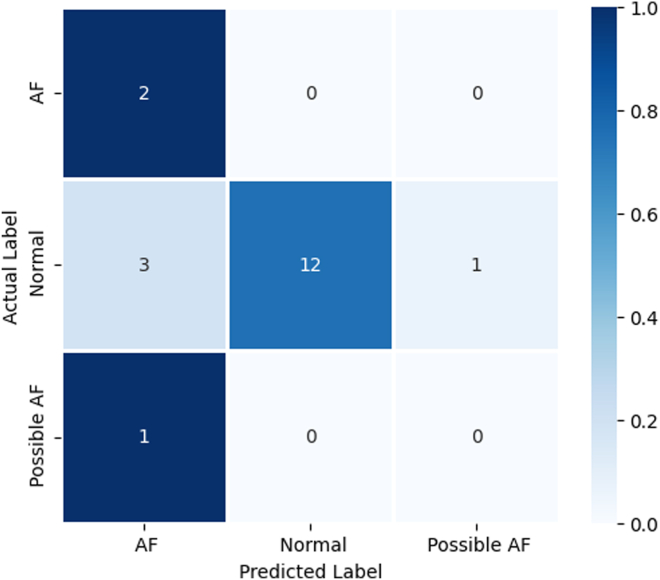
Confusion matrix showing the performance of our approach when predicting normal, possible AF, and AF electrocardiograms. AF = atrial fibrillation.

The 140 snippets were projected onto the existing latent space using the trained GTM, with the responsibilities for the ECG snippets relating to a single ECG aggregated to get 19 individual ECG probability distributions. From these, we then calculated the total responsibility for each macrocluster, with the ECG being assigned to the macrocluster with the highest value. Referring to Figure 6 for reference, an ECG was considered to be AF if the macrocluster it was assigned to was either 1 or 5 (the red or the orange cluster), possible AF if the macrocluster assigned was cluster 3 (the pink cluster), and normal if the macrocluster assigned was either cluster 2 or 3. Assigning the clusters this way and comparing the predicted label with the actual generated an overall accuracy of 74%. The full breakdown can be viewed in the confusion matrix in Figure 8.

### Visualizing an individual participant’s trajectory

**Figure 9:**
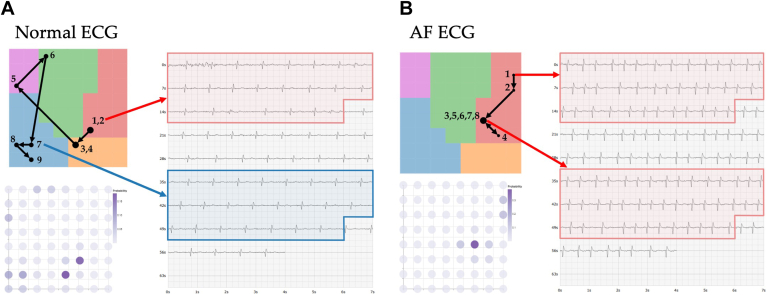
Displays the final output from our proposed methodology. Each ECG is projected onto the trained membership map to see where each snippet is assigned and how the model’s decision changes over time. A probability distribution is also produced alongside this projection to provide the user with the area of the membership map most responsible for generating that ECG. Alongside the projections, the actual PDF ECG is provided with the highlighted sections to allow the user to check the model’s cluster assignments. **A:** Shows the output for an ECG recorded from a participant diagnosed as normal. **B:** Shows the output for the ECG recorded on a participant diagnosed as having AF. AF = atrial fibrillation; ECG = electrocardiogram.

By collating the node locations associated with sequential ECG snippets derived from a single, continuous recording, and arranging them in chronological order, we enable the visualization of the temporal evolution of a participant’s ECG. The ECG snippet macrocluster assignment can then be validated by looking back at the original ECG PDF and evaluating the ECG between the start and end points of the snippet. Figure 9 shows this using the 2 test ECGs projected onto the macrocluster regions determined using the training data. The trajectory where the participant’s ECG was diagnosed as normal is presented in Figure 9A, with Figure 9B containing the trajectory where the participant’s ECG was diagnosed as AF. Alongside the membership map projections, we also show the overall probability distribution for ECG, displayed in the bottom left of Figure 9A and 9B. Given that GTM at its core is a probabilistic model, we can calculate for every point the probability that the data were generated from every latent node,^23^ essentially displaying the soft clustering performed by GTM. By averaging the probability distributions for each snippet from an ECG, we can generate a probability distribution per ECG to see which latent nodes were most likely to have generated it.

## Discussion

The methodological approach proposed in this paper has the potential to enhance decision making in AF screening by providing a comprehensive assessment and characterization of ECG signals, with a particular emphasis on specific regions within the membership map. By capturing subtle signal variations and analyzing their trajectories, our approach offers deeper insights into individual heart rhythm patterns that may be overlooked by traditional screening methods. This refined analysis facilitates a more accurate evaluation of electrophysiological abnormalities and improves risk stratification, enabling clinicians to identify high-risk patients more effectively.

Moreover, the insights gained from this method can support personalized treatment strategies, allowing for tailored interventions that align with the unique characteristics of each patient’s condition. This could be achieved by projecting new, unseen ECGs into the latent space, generating the appropriate visualizations, and matching their trajectory to similar known examples to identify successful treatment strategies that could be applied. Ultimately, this approach aims to enhance the overall effectiveness of AF detection and management, leading to improved clinical outcomes and more efficient use of health care resources. The visualizations generated using this approach convey far more nuanced detail than a binary positive or negative result and in turn hold significant promise for widespread community screening initiatives. For example, it could be seamlessly integrated into everyday environments such as supermarkets, where accessibility and convenience could drive early detection on a population-wide scale. By bridging advanced AI/ML methodologies (such as the one proposed) with clinical practice, we aspire to contribute to more effective detection of AF in diverse patient populations.

The traditional application of AI/ML in improving AF screening focuses on developing deep learning models, such as CNNs, to predict whether or not an ECG signal shows AF.^14^, ^15^, ^16^, ^17^, ^18^ These models perform this task very well, with areas under the curve ranging from 0.8^18^ to 0.87^17^ and accuracies up to 98.1%.^14^ Even though classification tasks addressed using these techniques have their merits, they can never be correct 100% of the time (as is the case with all ML models). The black-box nature of these approaches means that once a prediction has been provided, there is no real way to truly understand how and why the model has arrived at this decision, which in turn can affect the trust shown to the model by clinical experts. Researchers have attempted to interpret their models using post hoc approaches such as Grad-CAM^17^; however, this approach is known to highlight nonimportant areas of the input as important, potentially strengthening the distrust in the model.

Our approach directly addresses these issues through several key improvements. First, by presenting all model decisions in clear, interpretable visualizations, users can understand the rationale behind each decision. Second, we established a robust methodology for generating macrocluster regions with precise boundaries, enhancing the clarity and reliability of the model’s outputs. Third, we have shown that even though this methodology was not trained using diagnostic information, it was still able to classify the ECGs with reasonable accuracy. Even though the accuracy may not be in line with other metrics reported in the literature, these models were trained specifically for the task of detecting AF. In addition, our approach managed to classify all normal ECGs with 100% accuracy, which is crucial for a screening environment because it further builds trust in the approach while substantially reducing the number of ECGs that need to receive additional attention. Finally, our approach links each model decision back to the original data, providing users with a platform to override the decision if they deem it inaccurate, thus ensuring greater control and trust in the model’s outputs.

The work by Adeniji et al^19^ also addressed the issues outlined earlier using a fully interpretable multiple logistic regression model. Rather than generating a binary prediction, they prioritized ECG screening to reduce the workload on clinical experts. However, a notable limitation of their approach was that, despite analyzing long-form ECGs over several weeks, they required all ECGs to be collated before ordering could be performed. In contrast, our approach facilitates real-time application as ECG data are collected, enabling faster decision making and potentially improving screening accuracy. This immediacy in processing not only streamlines the workflow for clinicians but also enhances the overall efficiency of the screening process.

### Limitations

Our proposed methodology has several limitations that should be acknowledged and addressed through further research. The first limitation of our approach is that we used features extracted from the ECG snippets rather than the raw ECG signals. This feature extraction process can be tedious and introduces an additional layer of potential errors into the analysis. Another potential limitation lies in the GTM algorithm’s reliance on Gaussian distributions, which may struggle to accommodate outliers in the data. Consequently, this can lead to data being condensed into a smaller area of the latent space, potentially failing to capture the underlying relationships within the data adequately. Future analyses could address this issue by using t-GTM, an alternative to standard GTM that uses t-distributions, which are less sensitive to outliers.^34^

In addition, a limitation of our proposed methodology is that the number of macroclusters and the values of k used in the unsupervised KNN are selected manually. In addition, the demographic of the data collected and analyzed in this study is relatively small, comprising shoppers from Northwest England who use supermarkets for their purchases. To gain a comprehensive understanding of the benefits and generalizability of our approach, further testing would need to be conducted using data from a broader demographic. Finally, detection and confirmation of AF are only the first step of the clinical evaluation, characterization, and holistic management of AF,^35^^,^^36^ which are not addressed in our study.

## Conclusion

The methodology proposed in this study offers a promising advancement in AF screening by providing a nuanced approach to ECG analysis that extends beyond traditional classification models. By integrating interpretable visualizations and a robust clustering framework, this approach addresses some of the limitations of existing AI/ML models, such as the opacity and occasional unreliability of their decision-making processes. Unlike conventional deep learning models, which often present AF predictions in an unexplainable “black-box” format, our approach allows clinicians to observe model decisions transparently, supporting a clearer rationale for each diagnosis. Furthermore, the capacity for real-time analysis facilitates timely clinical decision making, optimizing the screening workflow and potentially improving patient outcomes.

Although this method demonstrates significant potential, we recognize limitations that future research should address, such as refining feature extraction methods, accommodating data outliers, and expanding testing across diverse populations. Nonetheless, this work marks a critical step toward bridging advanced ML techniques with clinical practice, ultimately aiming to enhance AF detection accuracy, foster trust among clinical experts, and support personalized, effective patient care.
