# Supplementary material for: AI-driven clustering and visualization of electrocardiogram signals to enhance screening for atrial fibrillation: The supermarket/hypermarket opportunistic screening for atrial fibrillation study
Source: Heart Rhythm O2. 2025 Jul 12;6(10):1601–12. doi: 10.1016/j.hroo.2025.07.003 (PMC12570188; doi:10.1016/j.hroo.2025.07.003)
Supplement: Supplementary Material [file mmc1.docx]

**Supplementary Material:**

**AI-driven clustering and visualisation of ECG signals to enhance screening for atrial fibrillation: The supermarket/hypermarket opportunistic screening for atrial fibrillation (SHOPS-AF) study**

Ryan A. A. Bellfield ^1,2^, Pablo Rendon Hormiga ^3^, Ivan Olier ^1,2^, Robyn Lotto ^2,4^,

Ian Jones ^2,4^, Gregory Y. H. Lip ^2,5^, Sandra Ortega-Martorell ^1,2,*^

^1^ Data Science Research Centre, Liverpool John Moores University, Liverpool L3 3AF, UK;

^2^ Liverpool Centre for Cardiovascular Science at University of Liverpool, Liverpool John Moores University and Liverpool Heart & Chest Hospital, Liverpool, UK;

^3^ Escuela Colombiana de Ingeniería Julio Garavito, Bogota 111166, Colombia;

^4^ School of Nursing and Advanced Practice, Liverpool John Moores University, Liverpool L2 2ER, UK;

^5^  Danish Center for Health Services Research, Department of Clinical Medicine, Aalborg University, Aalborg, Denmark


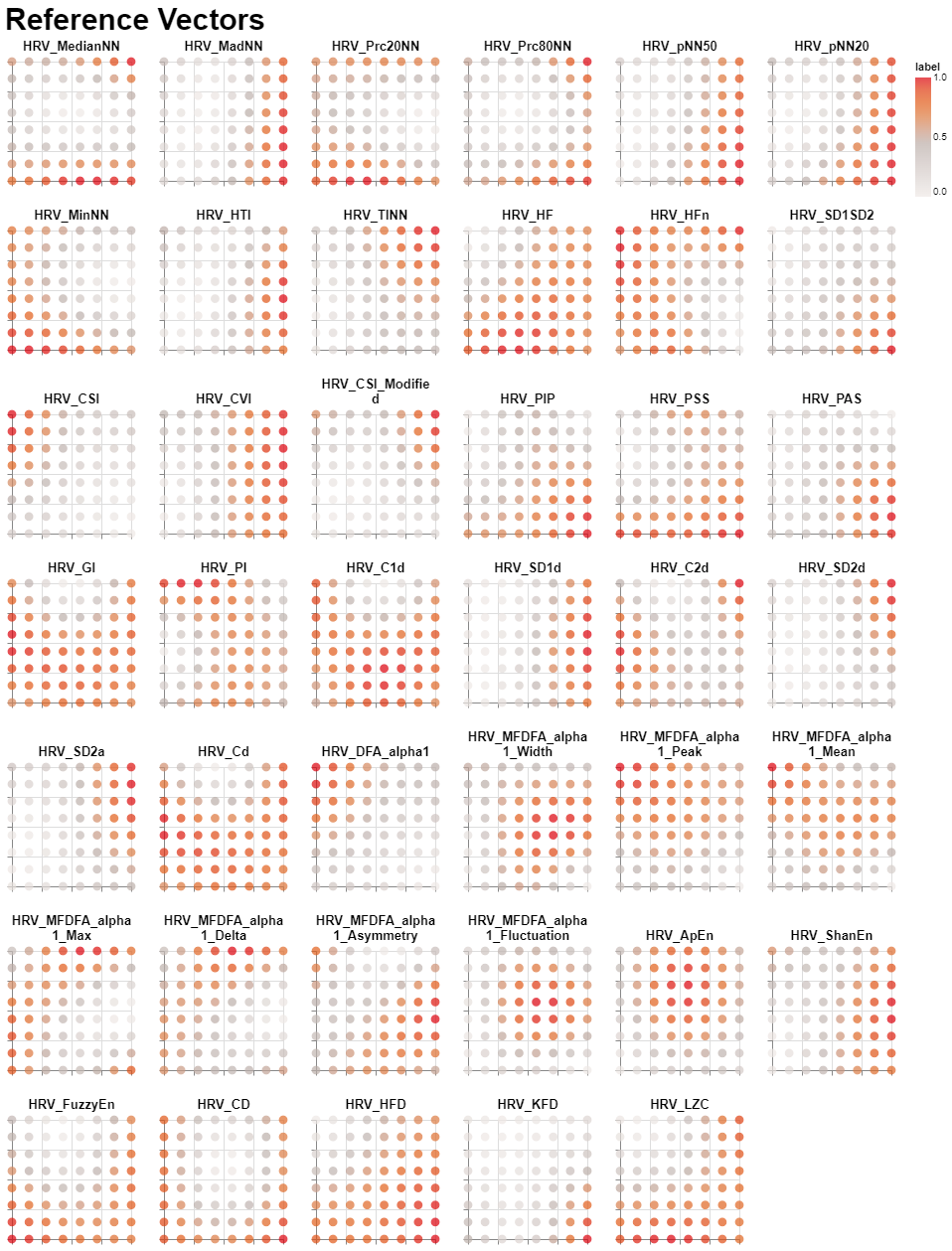


Figure S1. The reference maps for all the variables used to train the GTM model. These reference maps provide the user with key information about the models’ decision making, and how each variable affected the latent node data cluster assignments. For the variable names, please refer to table 1 in the main manuscript.
